# Acceptance of New Technology: A Usability Test of a Computerized Adaptive Test for Fatigue in Rheumatoid Arthritis

**DOI:** 10.2196/humanfactors.3424

**Published:** 2014-12-04

**Authors:** Stephanie Nikolaus, Christina Bode, Erik Taal, Harald E Vonkeman, Cees AW Glas, Mart AFJ van de Laar

**Affiliations:** ^1^ University of Twente Department of Psychology, Health & Technology Enschede Netherlands; ^2^ Radboud University Medical Center Expert Center for Chronic Fatigue Nijmegen Netherlands; ^3^ Medisch Spectrum Twente Department of Rheumatology and Clinical Immunology Enschede Netherlands; ^4^ University of Twente Department of Research Methodology, Measurement and Data Analysis Enschede Netherlands

**Keywords:** usability test, technology acceptance, computerized adaptive test (CAT), fatigue, rheumatoid arthritis

## Abstract

**Background:**

Little is known about the acceptance and usability of computerized adaptive tests (CATs) among patients with rheumatoid arthritis (RA). The main difference between completing a CAT and a traditional questionnaire concerns item presentation. CATs only provide one item at a time on the screen, and skipping forward or backward to review and change already given answers is often not possible.

**Objective:**

The objective of this study was to examine how patients with RA experience a Web-based CAT for fatigue.

**Methods:**

In individual sessions, participants filled in the CAT while thinking aloud, and were subsequently interviewed about their experience with the new instrument. The technology acceptance model (TAM) was used to structure the results.

**Results:**

The participants were 15 patients with RA. They perceived the CAT as clear, brief, and easy to use. They were positive about answering one question per screen, the changing response options, layout, progress bar, and item number. There were 40% (6/15) of the participants that also mentioned that they experienced the completion of the CAT as useful and pleasant, and liked the adaptive test mechanism. However, some participants noted that not all items were applicable to everybody, and that the wordings of questions within the severity dimension were often similar.

**Conclusions:**

Participants perceived the “CAT Fatigue RA” as easy to use, and also its usefulness was expressed. A 2.0 version has been improved according to the participants’ comments, and is currently being used in a validation study before it will be implemented in daily clinical practice. Our results give a first indication that CAT methodology may outperform traditional questionnaires not merely on measurement precision, but also on usability and acceptance valuation.

## Introduction

### Innovative Technology and Health Care

The use of Web-based technology to monitor disease course and quality of life of patients will increase tremendously in the future due to demand for greater transparency in health care and innovations in the use of Web-based measurement technology. At least for patient reported outcome measures, patients themselves will directly use this technology, and information on technology acceptance is therefore of crucial importance to estimate the benefits and long-term effectiveness of innovative technology in health care [1]. The availability of this modern technology enables computer adaptive testing (CAT), but little is known about the impact of Web-based measurements and CAT on patients, and how they experience their use. This study investigates the usability of a Web-based computer adaptive test for fatigue in rheumatoid arthritis from the patients’ perspective.

Rheumatoid arthritis (RA) is a chronic auto-immune disease that is characterized by inflammation of the joints [2]. Typical symptoms are pain, fatigue, tender and swollen joints, stiffness, and functional limitations. Many patients report fatigue as being an annoying symptom with far-reaching consequences for daily life on a physical, emotional, and social level [3-6]. Several circular and interdependent processes between disease processes, cognitive/behavioral, and personal aspects are probably responsible for the occurrence of fatigue [7,8]. However, causal pathways are still unknown and no standard treatments are as yet available [9,10]. To gain more insight into its aetiology and treatment options, it is essential to be able to accurately measure fatigue in RA. Existing fatigue questionnaires have several disadvantages, for example, containing generic fatigue items that might be confounded by disease specific disability or disease activity, or being unidimensional, which is not in line with the patients’ experience. Usual questionnaires have a traditional, fixed-length format. Consequently, patients may feel that questions do not match with their individual level of fatigue or are redundant. Therefore, we developed a computerized adaptive test (CAT), which was based on the perspectives of patients with RA [11].

### Computer Adaptive Testing

In a CAT, items are successively selected from a large item bank, based on the patient’s previous answer. Measurement is thus tailored to the individual level, leading to greater measurement precision, with need of fewer items than traditional questionnaires [12]. For the construction of a CAT, an item pool has to be scaled using item response theory (IRT). With this method, item characteristics can be estimated for each item independently [12], and items can be placed on a continuum, ranging from no fatigue to severe fatigue. This information is required to ideally match the items to the patient’s previous answer, and ensures interindividual comparisons, even if patients filled in different items.

The CAT Fatigue RA has been constructed with multidimensional IRT [11], and consists of 196 items and three dimensions of fatigue (severity, impact, and variability). It provides separate estimates of each fatigue dimension, and the cross-information gained from items of correlated dimensions facilitates the selection of the next most informative items, and the final estimation of fatigue with optimal precision [13]. With this innovative method, measurement of fatigue in RA has become much more precise, and at the same time, more user friendly.

However, relatively little is known on how patients experience the use of CATs in the measurement of patient reported outcomes (PRO)’s. A few previous studies have shown that the overall user acceptance was quite high. Participants mainly expressed criticism on layout issues [14,15], and about half of the participants rated the assessment as useful [16]. However, these results are difficult to generalize due to differences between the CATs and the study designs. The aim of the present study was to examine how patients use and experience the CAT Fatigue RA.

**Figure 1:**
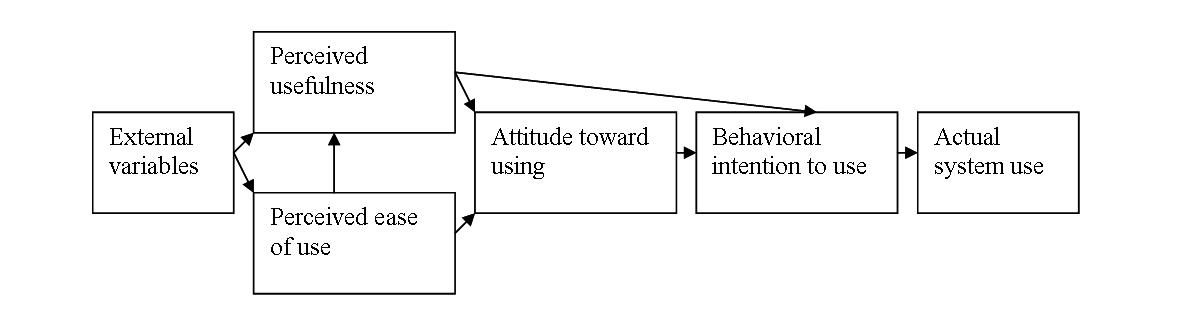
Technology acceptance model.

We were especially interested in whether patients would face any problems while filling in the CAT, and whether they would perceive it as a useful instrument. These aspects of usability are properly included in the Technology Acceptance Model (TAM) [17]; Figure 1 shows this model.

The TAM explains user acceptance of new technology by two main determinants: (1) perceived usefulness (PU), and (2) perceived ease of use (PEOU). PU refers to the degree to which a person believes a system to be worth using, for example, advantageous. PEOU refers to the degree to which a person believes that using a system does not cost much effort. Davis [17] suggests that the easier the use of a system is perceived, the higher the probability is that a person experiences the system as useful, and subsequently is willing to use it. Over the last decades, the TAM has been widely applied and has demonstrated its ability as theoretical model to guide understanding and explanation of technology acceptance [18]. An important model extension is the concept of perceived enjoyment [19], defined as “the extent to which the activity of using the computer is perceived to be enjoyable in its own right, apart from any performance consequences that may be anticipated” [20]. All variables together explain the attitude toward using a new technology, the behavioral intention to use it, and finally its actual use.

For the actual use of the CAT Fatigue RA, it is important that patients will not face any difficulties during its completion. The CAT is an Internet application that is intended to be used for PRO-monitoring in daily clinical practice and for research purposes. Patients gain access via their personal accounts of the Web-based Rheumatology Online Monitor Application (ROMA) that is used by many Dutch rheumatology units. Usually patients complete questionnaires on the Internet at home before their consultation at the rheumatology outpatient department. If a patient perceives filling in the CAT as difficult, not useful, or not enjoyable, the risk of drop out will be high.

Although many patients are already used to computer-based questionnaires, the completion of a CAT differs from filling in traditional fixed-length questionnaires. In a traditional questionnaire, patients see all questions immediately, and they have the opportunity to reread and to change answers. In contrast in a CAT, only one item at a time is provided on the screen, and often patients cannot skip forward or back [21]. This also means that patients cannot see how many or which items they already filled in and how many and which items are still left to complete.

In this study, patients filled in the CAT Fatigue RA in individual sessions while thinking aloud, and were subsequently interviewed on their experiences with the new instrument. Think aloud is a highly recommended method used to identify possible problems in measurement tools [22]. By combining the methods of think aloud and targeted interview questions, we aimed to identify all difficulties that patients could face while filling in the CAT. The TAM model served as guideline to report the results.

## Methods

### Participants and Procedures

Participants were selected from a sample of patients that had participated in a previous study [11]. All participants who had indicated interest in the results of the study received a thank-you letter with information on the study outcomes. At the end of this letter, patients were informed on future studies, and that they could register per email for participation. In case of registration, the patients received an email with detailed information on the new study, and were asked for agreement to receive a telephone call to make an appointment for an individual session with the first author. The ethical review board of the University of Twente approved the study.

Except for one appointment at a participant’s home, all sessions took place at the university. After receiving information on the study (eg, not the person, but the application will be tested), participants signed an informed consent and filled in some background questions. Then participants filled in the CAT while thinking aloud. In case a person forgot to articulate his or her thoughts, the researcher reminded him or her to do so. Finally, a brief interview on the CAT took place. The think aloud sessions and the interviews were recorded on audiotape. The travel costs for the participants were refunded.

### Measures

#### Background Information

Participants answered background questions (gender, age, education, and work status), and gave disease-specific information (disease duration, comorbidity, numerical rating scale; NRS, global health, pain, and fatigue). The NRSs had eleven points (ranging from 0 to 10) and the following anchors, very good/very poor, no pain/unbearable pain, no fatigue/totally exhausted.

#### Computer Adaptive Test Fatigue Rheumatoid Arthritis

**Figure 2:**
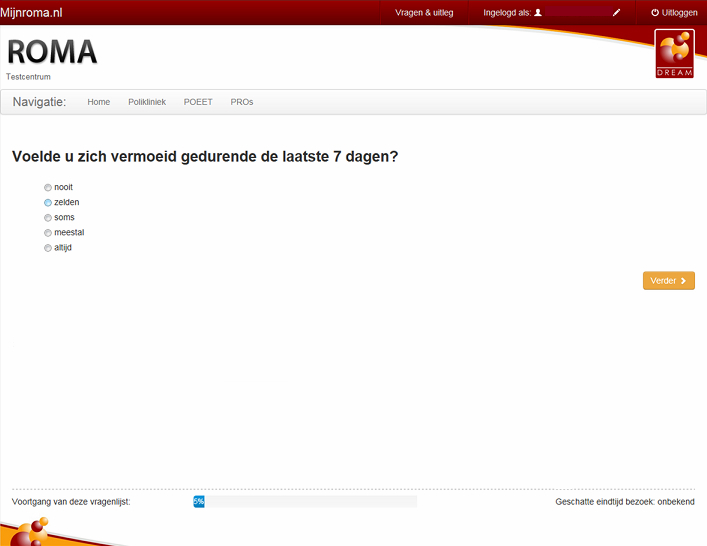
Screenshot of the CAT Fatigue RA.

The CAT item bank consists of 196 items and three dimensions; severity (13 items, example, *Did you feel tired during the last 7 days?*), impact (169 items, examples, *Have you felt down or dejected because of fatigue? During the past 7 days, I was too tired to do my most important tasks.*), and variability (14 items, example, *How did your fatigue change during the last 7 days?*). Each participant answered 20 questions, after which the CAT stopped automatically. It started with two random start-items per dimension, and always administered at least five items per dimension. These characteristics of the CAT were based on previous simulations in approximately 1000 virtual patients. This combination of numbers of items was found to be the most optimal solution in terms of test-length and measurement error on each dimension. Figure 2 shows an example screenshot of the CAT. The item with its response options is presented in the center of the screen. After answering the item, the patient can get to the next item by clicking on the yellow button. The blue bar shows the progress of the CAT administration by informing the patient which percentage of items is already filled in. The progress bar is part of the ROMA system and could be implemented because the CAT Fatigue RA has a fixed length of 20 items.

#### Thinking Aloud

The method of think aloud is the most prominent user-based usability method [22]. Participants are asked to state directly what they think, while completing a certain task using an application. This gives immediate insight into cognitive processes; whereas retrospective reports on thought processes imply the danger of losing information due to censoring and distortion. The usability tester should intervene with the participants thought process as little as possible. However, it is accepted to remind a participant to keep talking. Furthermore, it is important to conduct a think aloud study with a representative subject sample, meaning those people who will finally use the application. The think aloud approach is a good method to identify usability problems. It can detect deficiencies of the system, and also provides insight into the reasons why users experience certain deficiencies as a problem [22].

#### Interviews

After completing the CAT Fatigue RA, participants were asked about their experience with, and opinions on, the new measurement instrument, according to the interview scheme shown in Textbox 1.

**Interview scheme. CAT: computerized adaptive test.**

1. How did you experience completing the instrument?
2. What do you think about the successive administration of only one item per screen?
3. Did you notice that the response formats changed? What do you think about that?
4. How well could you read the questions? What do you think about letter size, colors, etc?
5. Did you notice the progress bar? What do you think about it?
6. What do you think about the length of the test/the number of questions?
7. Do you have any further comments about the CAT, did you notice anything else?

#### Analyses

The audio material (think aloud sessions and interviews) was transcribed verbatim. The interview material was sorted per interview question. The comments from the think aloud part were sorted per participant. To thoroughly analyze the data, a code scheme was developed in a combination of bottom-up (search for meaningful units in the transcripts) and top down (guided by TAM) methods by reading the transcripts in detail [22]. For each interview question, and for the think aloud material, topics with subcodes were identified and assigned to the transcripts. Topics that were mentioned in the interview material, as well as in the think aloud material, were not coded in the interview material, but reported together with the think aloud material to prevent double codes. The coding process was conducted in consensus between the first two authors.

## Results

### The Participants

There were six men and nine women diagnosed with RA that participated. Mean age was 56.13 years (SD 10.82) and mean disease duration was 12.40 years (SD 7.18). An overview about the participants and further patient characteristics are shown in Table 1.

The results of the think aloud sessions and the interviews will be reported in terms of the TAM and illustrated by quotes.

**Table 1:** Overview of participants.

Participant	Gender	Age, years	Education	Work	Disease duration, years	Comorbidity	NRS health^f^	NRS pain^g^	NRS fatigue^h^
1	F^b^	45	High^c^	Full-time	24	Yes	3	2	7
2	F^b^	49	High^c^	Full-time	15	Yes	7	5	6
3	F^b^	46	High^c^	Part-time	20	No	7	7	7
4	F^b^	66	Moderate^d^	Retired	6	No	4	6	7
5	F^b^	69	Moderate^d^	Retired	23	No	2	1	3
6	M^a^	59	High^c^	Part-time	10	No	6	4	3
7	M^a^	54	Moderate^d^	Disabled	10	No	5	5	8
8	F^b^	63	Low^e^	Household	5	Yes	4	4	5
9	F^b^	62	Moderate^d^	Retired	13	No	5	6	9
10	M^a^	71	Low^e^	Retired	3	No	0	0	1
11	F^b^	60	High^c^	Full-time	23	Yes	2	1	6
12	F^b^	63	Low^e^	Household	7	No	7	8	8
13	M^a^	60	Low^e^	Full-time	11	No	3	2	5
14	M^a^	40	Moderate^d^	Disabled	12	No	6	6	7
15	M^a^	35	Moderate^d^	Disabled	4	No	3	6	5
Mean		56.13			12.40		4.27	4.20	5.80
SD		10.82			7.18		2.12	2.46	2.18

^a^M=male

^b^F=female

^c^High, more than 14 years of education

^d^Moderate, 13-14 years of education

^e^Low, 12 or less years of education

^f^NRS health, 0 = very good and 10 = very poor

^g^NRS pain, 0 = no pain and 10 = unbearable pain

^h^NRS fatigue, 0 = no fatigue and 10 = totally exhausted

### Perceived Ease of Use

There were 80% of the participants (12 out of 15) that said that they experienced the CAT as clear and/or easy to complete. There were 87% of the participants (13 out of 15) that regarded it as advantageous to fill in only one question per screen, as it improved clarity. They found this presentation of items clear and well organized, making it easier to concentrate on the question and being really engaged in answering it. It was argued that too many questions at the same time can be overwhelming or cluttered, and with more simultaneous questions, people have the tendency to look ahead at the next question.

Quite clear, good. Yes, because of course you shouldn’t let yourself be tempted to read all the questions as quickly as possible because that is a mistake people often make, that they just immediately do everything, and then they maybe do not give a truly orientated answer.

Participant 7

Only two of the 15 participants reported that the presentation of only one item per screen did not really matter to them.

However, concerns also emerged related to this way of item presentation. In two thirds of the sessions, the CAT selected three or four of the following items of the dimension severity, Item 2, *During the last 7 days I felt tired.*; Item 3, *During the last 7 days I felt fatigued.*; Item 5, *Did you feel tired during the last 7 days?*; and Item 8, *Did you feel fatigued during the last 7 days?.* Most of the concerned participants (7 out of 10) were wondering whether the CAT provided the same question more than once, because to them the items looked very much alike. Due to the fact that it is not possible to scroll forward or backwards in the CAT, they became confused whether they had previously answered the item or not, and two of the seven participants indicated that they felt not able to answer in a consistent way.

All but one participant recognized that not all items had the same response options. There were 40% of the participants (6 out of 15) that said that they did not mind, it was no problem, and it did not distract them. There were two thirds of the participants that mentioned that it was advantageous that not all items had the same response options. In this way, items and response options match well with each other, which improves clarity. It was also argued that changing response options prevents people from always giving the same answer. Only one participant mentioned that it can be difficult to switch from one response format to another, however, this participant also reported having learned to fill in this kind of questions without thinking about them too long. All participants described the readability of the questions as clear, good, or comfortable to look at.

Regarding test-length, all participants were positive. They reported that the CAT was quick to complete, and they experienced the CAT as a clear and brief instrument, also in comparison to other measurement instruments. Moreover, for 40% of the participants (6 out of 15), the number of questions turned out smaller than they had expected. In general, participants described the test-length of the CAT as great, clearly better than expected, brief, or to the point.

### Perceived Usefulness

There were 40% of the participants (6 out of 15) that declared that they regarded the CAT as useful, for example, one participant considered the CAT to be a nice questionnaire with relevant questions.

(...) they are relevant questions, they are also much more focussed and clear questions, so I think it is a nice questionnaire (...) it really goes into fatigue and in a good way. So yes, I found it surprising, a surprising thing to do. Then it gives me more the notion that it is worthwhile to complete. You can enter, I was tired lately, the last 7 days, yes, the last month, but that says so little about fatigue.

Participant 11

There was one person that reflected on the adaptive testing mechanism.

What I really noticed was that if I had completed a question, that the computer sometimes took longer to get to the next question, and then I think, yes, that is logical, because then it is choosing the next question after all. (...) they are going to select which question fits with the answer to your previous question. I found it quite pleasant this way.

Participant 5

This person was of the opinion that the questions were useful, having good response options. There were 20% of the participants (3 out of 15) that criticized that not all questions were applicable to each patient (eg, being too fatigued to do voluntary work).

### Perceived Enjoyment

Completing the CAT, and the successive administration of only one item per screen, was experienced as pleasant, nice, great, excellent, or positive by two thirds of the participants. Furthermore, 40% (6 out of 15) described the progress bar as pleasant, great, useful, or comforting. There were 53% of the participants (8 out of 15) that liked the possibility of estimating their progress in completing the CAT; mostly the progress bar was recognized immediately.

A participant described it as pleasant that it did not take a lot of time to fill in the CAT. There were one third of the participants (5 out of 15) that were said to be glad with research into fatigue, and liked to support it through their participation. There were two participants that noted that it had been pleasant to fill in the CAT.

Please continue this because it is very nice. (...) I found it very nice after many years’ experience with ROMA and especially with the paper questionnaires. In the past I occasionally completed one of those things every two months. And then you think, aaaach, you really get to take such a pile of homework with you. So, no, it was very nice.

Participant 11

There were two other participants that also reported enjoying the idea that the CAT is testing adaptively.

To provide an overview about the different topics that are inherent to the use of a CAT in relation to those that are also inherent to Internet questionnaires in general, we summarized the main results in Table 2.

**Table 2:** Usability topics and their specificity to CAT.

Topic		Participants, N=15, n (%)	CAT specific/Internet fatigue measurement
Clear and/or easy to complete		12 (80)	Concerns CAT and Internet measurement
**One question per screen**			CAT specific
	Advantageous	13 (87)	
	Did not matter	2 (13)	
**Similar formulated items in two thirds (N=10) of the administrations**			CAT specific
	Confusion	7 (70)	
	No comment	3 (30)	
**Different response options, N=15**			CAT specific
	Positive opinion	15 (100)	
Good readability		15 (100)	Specific to Internet measurement
Good test-length		15 (100)	Concerns CAT and Internet measurement
Usefulness		6 (40)	Concerns CAT and Internet measurement
Criticism about not applicable items		3 (20)	CAT specific
Enjoyment		14 (93)	Concerns CAT and Internet measurement

## Discussion

### Usability of the Computer Adaptive Test

This study investigated the usability of the first version of the CAT Fatigue RA in a sample of its end users. Overall, the CAT was positively evaluated. It was described as easy to use, clear, and brief. Also some participants reported to perceive the CAT as a useful instrument, and appreciated the idea of the adaptive test mechanism. Participants reported pleasure while filling in the CAT. However, usability problems were also identified regarding similarity between items and the general applicability of some items.

Several elements are important for acceptance of new technology and actual use of a system. The perceived ease of use of the CAT was supported by this study. All participants described reading the questions as clear and good, and were positive about test-length. They said that the CAT was quick and easy to complete. Moreover, it was argued that changing response options prevents people from always giving the same answer. Nearly all participants appreciated the successive presentation of one item on the screen at a time, as it improves clarity and makes it easier to concentrate on the question.

The item presentation in the CAT gave participants less control during completion than they would have had while filling in a traditional questionnaire. Since there is no opportunity to skip forward or backwards, it is impossible to see all questions at the same time, answer them in a flexible order, or review and change already given answers [21]. Our results, however, give a first indication that end-users might experience filling in one item at a time as an advantage of a CAT.

### Item Formulation

Regarding item formulation, participants reported that four items in the severity dimension were formulated in a very similar way. As they could not skip back within the CAT, they were wondering whether the CAT presented items twice. To prevent a person feeling confused by these items while filling in the CAT, the first version of the CAT was adapted. Before the start of the instrument, a brief introduction has now been included. Therewith, patients are informed that some items may seem similar. In this way, it should be prevented that people will become distracted from filling in the CAT attentively, or that they might feel uncomfortable because they feel unable to answer in a consistent way. Another solution of this usability problem might be a more sophisticated algorithm that is able to recognize similar items, and consequently would avoid presenting them within one administration. However, then, usability issues might conflict with the selection of the best item in psychometric terms.

### Useful Instrument

Nearly half of the participants reported to perceive the CAT as a useful instrument. They emphasized that the CAT contained relevant and clear questions that cover patients’ fatigue experience. Furthermore, participants liked the idea of the adaptive test mechanism, and to receive items matched to their individual level of fatigue. Since participants were not explicitly asked about the usefulness of the CAT, this result is of special interest. Probably a higher percentage of participants had supported the usefulness of the CAT if a precise question about this topic had been included in the interview scheme.

However, some participants mentioned that not all questions were applicable to every patient. As a consequence, the response option “not applicable” was added to six items in the next version (eg, items about the impact of fatigue on work, cooking, or driving the car). When the “not applicable” option is chosen, the CAT receives no information for the fatigue estimation through this item, and will select the next optimal item for that particular patient as a substitute. In general, a comparable method might also be used to enable a skip forward function in a CAT. This could be useful in situations where it is adequate to give patients the possibility to skip questions they do not want to answer, for example, regarding private information. However, then an adequate way would be needed to communicate the option to skip items to patients without stimulating them to actually do this. Otherwise, too much loss of information might be the consequence. In the CAT Fatigue RA, a skip forward option does not seem necessary since the item pool has carefully been developed with a Delphi approach [23], and none of the participants of this usability study indicated the wish to leave an item unanswered.

Technology acceptance is also related to perceived enjoyment. Participants perceived completing the CAT as a pleasant experience. They enjoyed answering successively one item on the screen at a time, and liked the progress bar, as it informed them on their completion progress. Other positive remarks explicitly referred to the idea that the CAT is testing adaptively.

### Conclusions

The TAM turned out to be an adequate guideline to study the usability of the CAT Fatigue RA. Most participants reported to perceive the CAT as easy to use, and nearly half of the participants expressed that they perceived the CAT as useful. Perceived usefulness is of special importance for acceptance and use of new technology, and might be partly explained by the perceived ease of use [17]. We also found evidence for the role of perceived enjoyment [19] in this study. The combination of perceived ease of use, usefulness, and enjoyment, point to good acceptance and use of the CAT Fatigue RA when administered via the ROMA system in daily clinical practice in the future. Previous studies also reported a satisfactory acceptance of CAT in health care [14-16]. Furthermore, our results give a first indication that CAT methodology may outperform traditional questionnaires not merely on measurement precision, but also on usability and acceptance valuation.

This usability test provided important insights for further research with CATs. Similar formulated items and items that might not be applicable to each participant are typical issues that may be faced when implementing a CAT technology into practice. From a theoretical viewpoint, it is beneficial to include as many items as possible in the CAT item bank. Items that are similar to each other may also be useful, as they can be selected to measure very precisely at a certain level of fatigue. However, for the user, this rationale is not always clear, and may lead to usability problems. The same applies to an item bank with items that have no “not applicable” options. CAT was originally developed for educational and assessment purposes, where “not applicable” options are not appropriate. Adopting this technology into the health care context poses new usability questions.

This study has shown that the technology of CAT was well accepted by those who are intended to use it. The method of thinking aloud in combination with a consecutive interview on the participants’ experiences with the CAT has proven to be effective in uncovering usability problems, and thereby provided the opportunity to further improve the CAT. However, it cannot be ruled out that only those patients registered for the study who were already relatively familiar with using the computer. A small group of patients without computer experience [24,25] might possibly perceive using the CAT as less easy, less useful, and less enjoyable than the current sample that was between 35 and 71 years of age. Furthermore, in the think aloud part of the study, some participants had difficulties in distinguishing between issues regarding the CAT, issues on item level, and their personal situation. Some participants wanted to tell their personal stories, while others experienced the CAT as so easy to fill in that they could hardly find anything to comment on. This might be explained by the fact that many of the participants were already used to computer-based questionnaires. That no comments emerged on layout issues might also be related to the participants’ familiarity with ROMA, and the fact that it is a well established Web application that has already been in use for many years. The CAT runs in the same Web environment as ROMA, using the same colors and letter types. This points to the importance of layout familiarity in broad use of computer-based testing.

A possible field for future research on the CAT Fatigue RA is the development of a CAT version with a flexible stopping rule that ends the item administration in cases when a certain measurement precision is reached before 20 items have been administered. This could lead to even more efficient measurement. However, the realization is challenging because the standard error on the separate dimensions does not always decrease monotonously as in a unidimensional CAT. Such nonmonotone progress of the standard error is inherent to the multidimensional CAT algorithm that takes information into account of all three dimensions at the same time. Future research should make clear which possibilities are available for our CAT regarding a flexible stopping rule.

To conclude, the CAT Fatigue RA turned out to be perceived as an easy and useful measurement instrument that was also enjoyed by participants. This study provided insight into usability problems, leading to adaptations to the CAT. Moreover, participants described usability aspects that exceed traditional questionnaires. Next questions concerning the usability of CAT methodology are related to attractiveness of adaptive measurement in the long run. It is possible that the initial enthusiasm for this innovative measurement instrument will decrease when patients use the CAT on a regular basis and also for different purposes. However, there is a good chance that CATs will remain attractive since patients receive different items each time, which prevents boredom and predictability. However, it might also be imaginable that those different items provoke scepticism about the comparability of CAT scores of repeated measures and/or between persons. These topics have to be examined in detail in future research.

## Abbreviations

CAT: computer adaptive testing/computerized adaptive test
IRT: item response theory
NRS: numerical rating scale
PEOU: perceived ease of use
PRO: patient reported outcomes
PU: perceived usefulness
RA: rheumatoid arthritis
ROMA: Rheumatology Online Monitor Application
TAM: technology acceptance model
